# Primary care management of mild cognitive impairment in a stroke survivor: A case report on facilitating return to work

**DOI:** 10.51866/cr.731

**Published:** 2024-12-26

**Authors:** Afiza Hanun Ahmad @ Hamid, Nor Shahrina Mohd Zawawi, Mohd Fairuz Ali, Noor Azah Aziz

**Affiliations:** 1 MD, MMed (Family Medicine), Klinik Kesihatan Sitiawan, Jalan Datuk Ahmad Yunus, Sitiawan, Perak, Malaysia. Email: dr.afizahanun@moh.gov.my; 2 BSLT, MSLT, PhD (Stroke Rehabilitation), Department of Medical Rehabilitation Services, Hospital Canselor Tuanku Muhriz (HCTM), National University of Malaysia (UKM), Kuala Lumpur, Malaysia.; 3 MBBS, MMed (Family Medicine), Department of Family Medicine, Medical Faculty, Hospital Canselor Tuanku Muhriz (HCTM), National University of Malaysia (UKM), Kuala Lumpur, Malaysia.; 4 MD, MMed (Family Medicine), MPhil, Department of Family Medicine, Medical Faculty, Hospital Canselor Tuanku Muhriz (HCTM), National University of Malaysia (UKM), Kuala Lumpur, Malaysia.

**Keywords:** Return to work, Rehabilitation, Cognitive training

## Abstract

This case report delves into facilitating return to work (RTW) in a working-age stroke survivor. The patient was a 42-year-old Malay man who experienced multifocal lacunar infarctions in April 2022. He demonstrated substantial motor function recovery but presented with subtle cognitive deficits impacting various domains. The report outlines the diagnostic process of cognitive assessments and discusses the patient’s medical history and stroke-related factors. The management plan encompassed a multidisciplinary approach in primary care, which involved incorporating cognitive rehabilitation, discussing barriers and exploring the perception of the ability to RTW. This case underscores the intricacies of mild cognitive impairment in working-age stroke survivors and emphasises the need for tailored interventions in primary care to optimise cognitive recovery and enhance the overall quality of life.

## Introduction

The rising prevalence of ischaemic stroke among young people necessitates focused attention.^1,2^ Patients with mild stroke often lack medical follow-up and are expected to resume their prestroke professional lives without additional support.^3^ Stroke care now involves multiple components: acute care in stroke units, rehabilitation, transfer of care and long-term management in primary care settings.^4,5^ Family physicians play a crucial role in long-term post-stroke care by implementing proactive screening measures, conducting preventive interventions to prevent recurrent stroke and coordinating rehabilitation to optimise the quality of life. Effective post-stroke rehabilitation not only focuses on restoring motor function but also addresses cognitive recovery, facilitates communication and promotes return to work (RTW).^4^ Supporting RTW entails physicians considering various factors such as healthcare quality, socioeconomic status, physical and functional disabilities and cognitive impairment.^6^ However, cognitive impairment could be missed, as it is not regularly screened at the primary care level.^4^

This case report presents the case of a middle-aged man with multifocal lacunar infarctions caused by multiple cardiovascular risk factors, acutely managed in a hospital and later discharged to a primary care setting. The case highlights the need for a comprehensive assessment and coordination of interdisciplinary rehabilitation teams at the primary care level to facilitate RTW relative to the challenges caused by the presence of mild cognitive impairment - a critical aspect that is often overlooked in the assessment of young stroke survivors.

## Case presentation

A 42-year-old Malay man, who was working as a dental technologist and had underlying hypertension, type 2 diabetes mellitus and dyslipidaemia, was diagnosed with multifocal lacunar infarctions in 2022. He was an active smoker, consuming up to 10 cigarettes per day since adolescence, but denied any alcohol consumption.

The patient initially experienced left-sided weakness that improved within 24 hours and sought medical attention at a private clinic. Despite normal blood pressure and neurological findings, he was referred to a hospital for further management. However, he went to an outpatient clinic and was diagnosed with musculoskeletal pain.

A week later, the patient had a second episode of left-sided weakness and sought help from a health clinic. He was alert and well-built but displayed slurred speech, hemiplegic gait, facial asymmetry and left-sided hemiparesis. He was referred to a hospital, where computed tomography revealed recent infarctions in several brain regions, including the right anterior temporal lobe, right corona radiata, right basal ganglia and right occipital lobe.

He was treated for young stroke and showed motor function improvement over a month. He decided to RTW despite having a few days of medical leave left. Upon returning to work, the patient made errors with denture orders, leading to stress and reprimand. Realising his emotional needs, he sought treatment at the health clinic for low mood, frequent tearing and feelings of uselessness and unworthiness but without any suicidal thoughts. However, he refused referral to a counsellor or psychiatrist.

At the health clinic, the patient was assessed by a family medicine specialist (FMS) trained in dementia care, as his cognitive impairment met the criteria for mild neurocognitive disorder according to the DSM-5. On examination, the patient was alert and spoke in a low, monotonous voice with slurring. His vital signs and neurological findings were normal, with a motor strength of 5/5. Additionally, his blood test results were unremarkable. He was also noted to be independent in his activities of daily living (ADLs) based on his clinical findings and Modified Barthel Index. A post-stroke checklist (PSC) was also used to review the patient’s condition (Table 1).

**Table 1:** Post-stroke checklist.^7^

Item	Checklist (since your stroke or last assessment)	Patient’s assessment
Secondary prevention	Have you received any advice on health-related lifestyle changes or medications for preventing another stroke?	Yes
Activities of daily living	Are you finding it more difficult to take care of yourself?	No
Mobility	Are you finding it more difficult to walk or move safely from a bed to a chair?	No
Spasticity	Do you have increasing stiffness in your arms, hands and/or legs?	No
Pain	Do you have any new pain?	No
Incontinence	Are you having more problems controlling your bladder or bowels?	No
Communication	Do you find it more difficult to communicate with others?	No
Mood	Do you feel more anxious or depressed?	Yes
Cognition	Do you find it more difficult to think, concentrate or remember things?	Yes - Interfering with his work
Life after stroke	Are you finding things important to you more difficult to carry out (e.g. leisure activities, hobbies, work or relationships with loved ones, where appropriate)?	Yes - Difficulty in carrying out work
Relationship with family	Has your relationship with your family become more difficult or stressful?	No

Thereafter, the Montreal Cognitive Assessment (MoCA) was conducted, which indicated mild cognitive impairment, with a score of 24, highlighting issues with short-term memory, attention and concentration. The presence of clinical mood disturbance was also explored. There was no feature suggesting depression and post-stroke dementia, as the Patient Health Questionnaire-9 and Two Questions with Help Question scores were normal.^8,9^

The patient received care for stroke-related factors and rehabilitation. His antihypertensive and antidiabetic medications were optimised, and he underwent comprehensive post-stroke rehabilitation. An interdisciplinary team (IDT) consisting of an FMS, a physiotherapist and an occupational therapist, along with the patient and his wife, developed a comprehensive intervention plan (Table 2). The patient was allowed to work, as he could perform general and instrumental ADLs, with a recommendation for workplace modification (i.e. light-duty tasks excluding those involving denture moulding) until the next review. This strategy aimed to alleviate stress and foster a supportive environment for recovery. The IDT conducted virtual consultations to monitor progress, adjusted the plan as needed and supported his goal of returning to work.

**Table 2:** Interdisciplinary team discussions.

**Goals of therapy**
1. Resolve cognitive impairment. 2. Facilitate successful RTW. 3. Prevent post-stroke depression.
**Barriers identified**
1. The cognitive therapy sessions were difficult to intensify due to the long interval between sessions. 2. The patient refused referral to a counsellor or psychologist.
**Proposed and implemented solutions**
1. The cognitive therapy sessions and training included the patient’s wife, with the goal of continuing therapy at home. 2. A letter was sent to the patient’s superior to arrange work modifications until the next review, aiming to prevent mistakes and reduce distress. 3. An earlier appointment with the FMS was scheduled to assess the patient’s mental status and prescribe antidepressants if necessary.
**Components of cognitive rehabilitation and examples of activities**
1. Memory: Playing memory games and recalling games 2. Attention: Completing wooden puzzles and solving simple mathematical questions 3. Concentration: Playing word search and finding hidden things 4. Basic concept numbers: Flashing card numbers and matching numbers

The patient responded favourably to the rehabilitation goals and light-duty measures and gradually recovered from the psychological impact of the work-related incident. His MoCA score improved to 30/30 within 6 weeks of intervention. Consequently, he successfully returned to his work.

## Discussion

### Post-Stroke Checklist (PSC)

Unmet needs in stroke survivors may be overlooked in busy health clinics. Thus, a PSC was developed to help physicians identify patient needs beyond medical and visible disabilities and facilitate appropriate referrals.^7^ The PSC consists of 11 items on long-term problems, enabling the identification of additional support for survivors.^10^ Further investigation may be required to evaluate the feasibility and successful implementation of the checklist within individual settings, taking into consideration the service set-up.^10,11^

### Identification of post-stroke cognitive impairment

A systematic review of 12 cognitive screening tools found that the MoCA had superior sensitivity for detecting mild cognitive deficits after stroke over the Mini-Mental State Examination and other tools.^12^ This tool, available in various languages, can be administered quickly with minimal training. It consists of 30 items that evaluate several cognitive domains (Table 3), with a score of ≤26 indicating cognitive impairment.^13^

**Table 3:** Cognitive domains tested in the MoCA.

Domains	Skill Tested
Attention	Focusing, shifting, dividing and sustaining attention on a particular task
Memory	Visual/auditor)^r^ memory, working memory, episodic/semantic memory and procedural memory
Executive function	Planning, abstract thinking, organisation of thoughts, inhibition and conflict monitoring
Perception and praxis	Visuo-spatial ability, visuo-perceptual ability, unilateral neglect, inattention, apraxia, agnosia and prosopagnosia
Language	Aphasia

### Facilitation of RTW

Global cognitive impairment predicts successful RTW,^3^ making cognitive rehabilitation essential even in mild stroke cases. This rehabilitation involves direct remediation to reestablish previous behavioural patterns and compensatory strategy training to develop new cognitive patterns through internal or external mechanisms, such as aids or environmental adjustments. These strategies aim to restore impaired functions and help patients adapt to functional activities despite cognitive disabilities.^14^

Supporting stroke survivors in returning to work requires more than clinical activities. It may include identifying work barriers, performing work trials, adjusting work tasks, teaching specific work and self-management skills and coordinating with employers.^15^ Some RTW interventions involve collaboration with occupational therapists for workplace assessments,^16^ individual-specific adjustments and vocational counselling.^15^ However, there is limited high-quality evidence regarding the effectiveness of any specific interventions in promoting RTW among stroke survivors.^15^

In primary care settings, where rehabilitation support is limited, a multidisciplinary approach involving caregivers can significantly aid stroke survivors in learning and applying cognitive strategies. Engaging caregivers also makes them feel valued and able to discuss carer needs, helping to alleviate their caregiving burden.^17^ Conversely, exploring the perceptions of stroke survivors about their working ability and barriers, in addition to obtaining information about their cognitive capacities, is strongly linked to successful RTW.^18^ This information may help to gauge their motivation to resume work and identify areas needing further support to facilitate successful employment after stroke.

## Conclusion

This case report highlights the management of mild cognitive impairment following young stroke and addresses the necessity for a holistic and collaborative approach within primary care settings. Successful rehabilitation and positive RTW outcomes could be achieved by addressing cognitive impairment in stroke survivors through a multidisciplinary and individualised care approach.
